# Dean’s Advertisement of Lectures in the Cincinnati College

**Published:** 1838

**Authors:** Landon C. Rives

**Affiliations:** Dean; Cincinnati, Ohio


### ART. VIII.—MEDICAL DEPARTMENT OF THE CINCINNATI COLLEGE.

The session commences the last Monday of October and termiantes the last Saturday of February; but by a late regulation of the Faculty, four lectures will be delivered daily by the professors, during the period of examination of candidates for degrees, which will, *under no circumstances*, commence until the regular session shall have expired.

*Special and Surgical Anatomy*, by Joseph N. McDowell, M. D.

*General and Pathological Anatamy and Physiology*, by Samuel D. Gross, M. D.

*Surgery*, by Willard Parker, M. D.

*Obstetrics, and the Diseases peculiar to Women and Children*, by Landon C. Rives, M. D.

*Chemistry and Medical Jurisprudence*, by James B. Rogers, M. D.

*Materia Medica and Pharmacy*, by John P. Harrison, M. D.

*Theory and Practice of Medicine*, by Daniel Drake, M. D.

*Dissections and Practical Anatomy*, by Cary A. Trimble, M. D.

*Clinical Instruction* in the Cincinnati Hospital, by Doctors Drake, Parker and Rives.

Dr. Trimble will open the rooms for Practical Anatomy on the 1st of October; and Professor McDowell will commence, at the same time, a preliminary course of lectures on Osteology.

Expenses. Tickets of the Professors, $15 each. Matriculation fee $2. Library ticket, (which may be taken or omitted at the option of the student) $3. Hospital ticket $5. Ticket to the Anatomical rooms $10. (The two latter are required to be taken one session only.) Total $125.

Respectable boarding and lodging can be had for $3 per week.

Candidates for graduation are required to *study three years* under some respectable physician, to attend two full courses of lectures, one of which must be in this school; or to *have practiced medicine four years*, and to attend one full course in this school, before they will be admitted to an examination.

In the present deranged state of the currency, the Faculty deem it proper to announce that they will receive from students in payment of their fees, notes of reputable banks, belonging to the States in which they respectively reside, at par.

By order of the Faculty,

LANDON C. RIVES, Dean.

*Cincinnati, Ohio* 1838.

